# Clinical and functional outcomes associated with pulmonary complications after coronary artery bypass grafting

**DOI:** 10.1186/s13019-024-02538-9

**Published:** 2024-02-14

**Authors:** Altina Vitória Souza, Raquel da Cunha Carvalho, Daniela da Cruz Dias, Darley Gabrielle Teles Santana, Hayssa de Cássia Mascarenhas, André Luiz Lisboa Cordeiro, André Raimundo França Guimarães

**Affiliations:** 1 Centro Universitário Nobre, Red Bird Street, without number, Condominio Salvador Dali, house 47, Feira de Santana, BA Brazil; 2 Escola Bahiana de Medicina e Saúde Pública, Salvador, BA Brazil; 3 Instituto Nobre de Cardiologia, Feira de Santana, BA Brazil

**Keywords:** Cardiac surgery, Pulmonary function tests, Postoperative care, Functional performance, Muscle strength

## Abstract

**Introduction:**

Coronary artery bypass grafting(CABG) is a surgical treatment for coronary artery disease aiming at improving symptoms and life expectancy. Despite this, there are pulmonary and functional complications that may arise during the postoperative period due to invasive mechanical ventilation(IMV), cardiopulmonary bypass and immobility, leading to longer hospital stays.

**Objective:**

To evaluate the clinical and functional outcomes related to pulmonary complications in the postoperative period of CABG.

**Methods:**

Prospective cohort. During the ICU stay the patients were divided into: Non Complicated Group(NCG) who did not present complications and Complicated Group(CG) who presented complication. Functional variables were applied as the six-minute walk test(6MWT), gait speed, sit up and stand up test, Timed Up and Go, peripheral muscle strength, ventilatory, pulmonary function and Functional Independence Measure. These tests were applied preoperatively, at ICU discharge, hospital discharge and six months after surgery.

**Results:**

The study evaluated 90 patients, 59 in the NCG and 31 CG. In the 6MWT there was a 2%(*p* = 0.43) decrease in the NCG, while the decrease was 13%(*p* < 0.01) in the CG. In the MRC the drop was 2%(p = < 0.01) in the CNG, while in the CG the drop was 14%(p = < 0.01). In MIP the NCG had a 6%(*p* = 0.67) decrease, while the CG had a 16%(p = < 0.01) decrease.

**Conclusion:**

Patients with postoperative complications of CABG may have reduced functional performance, muscle strength, and pulmonary function at hospital discharge and after six months.

## Introduction

Coronary artery bypass grafting (CABG) is a surgical treatment for coronary artery disease that aims to improve symptoms and life expectancy in patients [1, 2]. Despite the benefits, there are pulmonary and functional complications that can arise during the postoperative period due to invasive mechanical ventilation (IMV), cardiopulmonary bypass and immobility, leading to longer hospital stays [3].

In recent years the number of CABG has increased significantly, becoming the most common procedure in cases of heart disease and widely performed for correction of valve disease, but its indication depends on the degree of comorbidities, age and clinical manifestations. However, in the year 2018 it was found more than a thousand deaths resulting from cardiac surgeries, among them CABG, considering the possible postoperative complications [4, 5].

Pulmonary complications after CABG are the most prevalent, and are associated with factors such as low preoperative pulmonary function, prolonged cardiopulmonary bypass, mechanical ventilation, and the presence of drains [6, 7]. These factors are associated with worse outcomes such as longer hospital stays, poorer quality of life, and worse sleep [7].

Because of the immobilism, pain and the patient’s clinical condition, functionality and balance are reduced in the postoperative period, thus evidencing that these patients can develop functional alterations and physical therapy treatment is necessary [8].

There are still few studies verifying the impact of these complications on clinical and functional outcomes in the short and long term. Considering this context, the aim of this study is to evaluate the clinical and functional outcomes related to postoperative pulmonary complications in patients undergoing coronary artery bypass graft.

## Methods

### Study design

This is a prospective cohort, carried out with patients undergoing CABG at the Instituto Nobre de Cardiologia em Feira de Santana, Bahia - Brazil, from January 2018 to February 2020.

### Inclusion and exclusion criteria

The following inclusion criteria were used: Individuals of both genders with Coronary Artery Disease (CAD), over 18 years of age, and undergoing coronary artery bypass grafting with cardiopulmonary bypass and median sternotomy. The exclusion criteria were valve disease, previous pneumopathy, those who did not understand how to perform the proposed techniques, those who presented hemodynamic instability during the evaluation, physical limitations such as amputation that compromised the performance of the exercises, and those who were unable to answer the questionnaires.

### Ethical aspects

Our study was submitted and approved by the Ethics and Research Committee of Nobre University Center at Feira de Santana – Bahia, Brazil obtaining opinion number 2.382.707. All participants signed an informed consent form.

### Outcomes

The primary outcome was functional performance. The secondary was cardiopulmonary bypass (CPB) time, mechanical ventilation (MV) time, length of stay in Intensive Care Unit (ICU) and length of hospital stay.

### Study protocol

In the preoperative period, clinical and surgical characteristics such as diabetes mellitus, hypertension, dyslipidemia, acute myocardial infarction, and sedentary lifestyle were collected. All these comorbidities were known through the medical records of each patient, with the exception of sedentarism, where the International Physical Activity Questionnaire (IPAQ) was applied in the long format, which evaluates 27 questions related to physical activities performed in a normal week, with light, moderate, and vigorous intensity, lasting 10 min continuously, divided into four categories of physical activity such as work, transportation, household activities, and leisure. Those who did not perform any physical activity for at least 10 min continuously during the week were considered to be inactive [10].

The next day, they all underwent a surgical procedure, were referred to the Intensive Care Unit (ICU) and, having been discharged, were sent to the inpatient unit. At all these times, they received routine care from the unit without any influence from the researchers. All patients were seen by the on-duty physical therapist and performed breathing exercises, orthostasis training on the first postoperative day, sitting in a chair, and walking on the second postoperative day when there was no clinical contraindication. All patients received pain relief with 1 g paracetamol 4 times daily for as long as necessary after discharge. The surgical procedure has always been carried out by the same surgical team.

During the ICU stay patients were divided into two groups: Non Complicated Group (NCG) which did not have complications during their ICU stay, and Complicated Group (CG) which had complications during their ICU stay. Complications included atelectasis, pleural effusion, pneumonia, pulmonary edema, severe respiratory distress, pulmonary embolism, pneumothorax, mediastinal infection, renal infection, arrhythmia, and coagulation disorder. The verification of complications was done through the medical record and/or in contact with the diarist doctor of the unit.

The groups were compared for cardiopulmonary bypass (CPB) time, mechanical ventilation (MV), length of stay in the Intensive Care Unit (ICU), and length of hospital stay. In addition, functional variables were applied such as the six-minute walk test, gait speed, sit and stand test, Timed Up and Go, peripheral muscle strength, ventilatory, lung function and Functional Independence Measure. These tests were applied preoperatively, at the time of discharge from the ICU, hospital and six months after the surgical procedure. The indication for discharge from the ICU was in accordance with the unit’s routine. Hemodynamic stability, no use of vasoactive drugs, no bleeding and no signs of infection were taken into account.

### Variables evaluated

The sit and stand test (SST) was performed through 5 repetitions, reproducing the act of sitting and standing up independently. During the test no devices or arms were allowed to be used for support. The arms should remain crossed over the patient’s pectoral to prevent use. It should be done as fast as the patient can, with a timer, without any encouragement from the evaluator [9].

In the Gait Velocity Test (GVT), the patient is instructed by the examiner to walk at normal speed on a 10-meter track. The examiner should take into account that the first two meters are for acceleration and the final two meters are for deceleration. Time will only be recorded between the second and the sixth meter [10].

In Time Up and Go (TUG) the patient must be seated on a chair with no arms, and must walk for a distance of three meters, return to the chair, and sit down again. The time is only timed from the moment the patient gets up from the chair and is paused when he or she sits down again [11].

The preoperative evaluation of the inspiratory muscle strength (Maximum Inspiratory Pressure (MIP)) was performed using an Indumed® analog manometer. During the evaluation it was requested a maximum expiration until the residual volume and then a maximum and slow inspiration until the total lung capacity, and this test was done through the method with the unidirectional valve, allowing a flow through an orifice of one millimeter aiming exclusion of the buccinator action, and repeated 3 times using the highest value reached since this value was not the last. The expiratory muscle strength (Maximum Expiratory Pressure (MEP)) was evaluated using the same device, and the patient was instructed to take a maximum inspiration until he reached his Total Lung Capacity (TLC), the mask was placed, and after that a maximum expiration was requested until the residual capacity was reached. The test was repeated 3 times and the result with the highest value was considered, but it could not be the last one [12].

For vital capacity evaluation a face mask was used, connected to the expiratory branch of the analog ventilometer (Ferraris - Mark 8 Wright Respirometer, Louisville, CO, USA) and the patient was instructed about all the test phases. The ventilometer was unlocked, zeroed, and soon after the face mask was placed on the subject’s face. The patient made a deep inspiration until reaching his total lung capacity (TLC), then a slow and gradual expiration until reaching his residual volume. After this, the ventilometer was locked and the result observed and noted. The test was repeated 3 times, considering the result with the highest value [13].

Peak expiratory flow was assessed using the Mini Wright® peak flow. During the evaluation, the patient was seated, with his head in a neutral position and a nose clip to prevent air from escaping through the nostrils. The patient made a deep inspiration up to full lung capacity, followed by a forced expiration with the mouth in the device. After three measurements, the highest value was chosen, and there could be no difference greater than 40 L between measurements [13].

The six-minute walk test (6MWT) assessed functional capacity, evaluation of response to intervention, risk of falling, and predictor of mortality. The application of the test was performed by a trained team, the volunteers were instructed how the 6MWT works, the test was performed in a corridor with a distance of 30 m flat, free and without obstacles for 6 min timed. After performing the 6MWT the patient sat in a chair and the following variables were monitored again: blood pressure, heart rate (HR), respiratory rate (RR), peripheral oxygen saturation (SpO2) through pulse oximeter, those who presented one of the variables outside the parameter did not participate in the test [14].

The Functional Independence Measure makes it possible to evaluate the capacity of the patient to execute the assigned tasks and the need for support from another person; the activities are divided into 18 items that make it possible to evaluate the independence of the patient in self-care tasks, motor and cognitive dimensions. To classify the level of independence of the patient, the score 1 was used for total dependence to perform the task and 7 for independence in the execution of the task, with a maximum value of 126 points [15, 16].

The Medical Research Council (MRC) evaluates peripheral muscle strength through the ability to overcome load in six muscle groups (shoulder abductors, elbow flexors, wrist extensors, hip flexors, knee extensors, and ankle dorsiflexors), scoring each group bilaterally from 0 to 5, where zero represents no contraction and five means the patient overcomes the maximum resistance imposed by the examiner. The minimum score for this test is 0 (tetraplegia), and it can reach up to 60 points (preserved muscle strength). A score of less than 48 may be suggestive of a polyneuromyopathy [17].

### Statistical analysis

For data analysis the SPSS program version 20.0 was used. Normality was assessed by the Shapiro-Wilks test. Continuous variables were expressed as mean and standard deviation. Categorical variables between groups were evaluated using Chi-square. For numerical variables the paired Student’s t-test was used. ANOVA was used to evaluate the variables preoperatively, at discharge and six months later. Paired Student’s t test comparing pre of the uncomplicated group with pre of the complicated group. Paired Student’s t test comparing discharge from the uncomplicated group with discharge from the complicated group. Paired Student’s t test comparing 6 months in the uncomplicated group with 6 months in the complicated group. It was considered as significant when *p* < 0.05.

## Results

The study evaluated 90 patients, with a mean age of 63 ± 5 years, 57 (63%) patients were male, in general they presented a high prevalence of sedentarism accounting for 68 (76%), the comorbidity most commonly found was SAH with 51 (57%) participants. The group with complications had a longer ICU stay (3 ± 2 days) than patients without complications, where, consequently, there was a difference in the length of hospital stay in patients without complications (8 ± 4 days) and with complications (14 ± 3 days). Table 1 shows the other characteristics of the patients.

**Table 1:** Clinical and surgical data of the patients studied

Variable	Total (n = 90)	No Complication (n = 59)	Complication (n = 31)	*p*
Age (years)	63 ± 5	62 ± 4	64 ± 5	0,63^a^
Gender				0,55
Male	57 (63%)	37 (63%)	20 (65%)	
Female	33 (37%)	22 (37%)	11 (35%)	
BMI (kg/m^2^)	25 ± 3	24 ± 2	26 ± 3	0,44 ^a^
Ejection Fraction	52 ± 5	52 ± 4	52 ± 5	0,84 ^a^
Level of Physical Activity				0,73 ^b^
Active	22 (24%)	15 (25%)	7 (23%)	
Sedentary	68 (76%)	44 (75%)	24 (77%)	
Comorbidities				
SAH DM DLP	51 (57%) 36 (40%) 26 (29%)	33 (56%) 23 (39%) 18 (31%)	18 (58%) 13 (42%) 8 (26%)	0,87 ^b^ 0,76 ^b^ 0,67 ^b^
Surgery time (hours)	4,4 ± 1,6	4,4 ± 1,9	4,3 ± 1,2	0,61 ^a^
Time of CPB (min)	91 ± 14	89 ± 12	92 ± 15	0,19 ^a^
Time of ICU stay (days)	3 ± 2	2 ± 1	4 ± 3	0,03 ^a^
Time of hospital stay (days)	11 ± 4	8 ± 4	14 ± 3	< 0,01 ^a^
MV time (hours)	7 ± 4	6 ± 3	7 ± 4	0,23 ^a^
Number of drains	2 ± 1	2 ± 1	2 ± 1	0,95 ^a^
Number of grafts	2 ± 1	2 ± 1	2 ± 1	0,94 ^a^

(a) Paired Student’s t-test; (b) Chi-square BMI - Body Mass Index; CPB – Cardiopulmonary bypass; SAH - Systemic Arterial Hypertension; DM - Diabetes Mellitus; DLP - Dyslipidemia; ICU - Intensive Care Unit; MV - Mechanical Ventilation

Table 2 shows the behavior of the variables associated with functional performance, lung function, and ventilatory muscle strength at the three moments of the study (preoperative, hospital discharge, and six months). We found that during the preoperative period both groups showed no statistical significance in relation to functionality and pulmonary function, however, both at hospital discharge and 6 months after surgery, there was a reduction in all variables evaluated of muscle strength and pulmonary function with statistical significance, except in Peak expiratory flow that had no significant decrease. On the 6MWT, there was a 2% (*p* = 0.43) decrease in the uncomplicated group, while the decrease was 13% (*p* < 0.01) in the group with complication. In the MRC the drop was also 2% (p = < 0.01) in the uncomplicated group, while in the group with complications the drop was 14% (p = < 0.01). In the MIP, the uncomplicated group had a 6% decrease (*p* = 0.67), while the group with complications had a 16% decrease (p = < 0.01).

**Table 2:** Data from the functional and pulmonary function variables of the groups studied

Variable		No complication (*n* = 59)					Complication (*n* = 31)													
	Preoperative	Discharge from ICU	Discharge	Six months	p^a^	Preoperative	Discharge from ICU	Discharge	Six months	p^a^	p^b^	p^c^			p^d^			p^e^		
Functional																				
6MWT (meters)	501 ± 99	399 ± 78	455 ± 89	495 ± 94	0,43	494 ± 101	345 ± 77	387 ± 77	432 ± 88	< 0,01	0,67		< 0,01	< 0,01		0.02				
WS (m/s)	1,5 ± 0,7	0,8 ± 0,4	1,1 ± 0,5	1,6 ± 0,6	0,12	1,5 ± 0,5	0.7 ± 0.4	0,8 ± 0,5	1,1 ± 0,4	< 0,01	0,87		< 0,01	0,07		0.43				
SST (s)	11,2 ± 0,6	9,5 ± 0,7	12,5 ± 0,8	11,4 ± 0,9	0,45	11,9 ± 0,7	8.9 ± 0.6	16,7 ± 1,6	15,9 ± 0,9	< 0,01	0,76		< 0,01	< 0,01		0.32				
TUG (s)	9,8 ± 1,1	11,3 ± 1,2	10,3 ± 1,2	9,9 ± 0,9	0,32	10,1 ± 1,3	13.5 ± 1.1	15,8 ± 2,1	14,2 ± 1,3	< 0,01	0,59		< 0,01	< 0,01		0.03				
MRC	58 ± 1	45 ± 3	50 ± 3	57 ± 2	< 0,01	58 ± 2	44 ± 2	43 ± 4	50 ± 3	< 0,01	0,79		< 0,01	< 0,01		0.78				
FIM	125 ± 1	115 ± 4	119 ± 2	124 ± 2	0,68	125 ± 1	111 ± 5	101 ± 4	115 ± 4	< 0,01	0,92		< 0,01	< 0,01		0.45				
Pulmonary Function																				
MIP (cmH2O)	104 ± 9	67 ± 11	89 ± 10	98 ± 8	0,67	102 ± 8	55 ± 11	71 ± 8	86 ± 9	< 0,01	0,59		< 0,01	< 0,01		0.03				
MEP (cmH2O)	92 ± 8	70 ± 10	79 ± 7	87 ± 8	0,51	93 ± 10	65 ± 9	66 ± 8	75 ± 9	< 0,01	0,78		< 0,01	< 0,01		0.12				
VC (ml/kg)	55 ± 6	38 ± 7	45 ± 4	52 ± 4	0,32	54 ± 5	32 ± 4	39 ± 4	45 ± 5	< 0,01	0,87		< 0,01	< 0,01		0.04				
PEF (L/min)	321 ± 77	256 ± 78	289 ± 67	302 ± 71	0,43	333 ± 71	220 ± 78	276 ± 56	321 ± 79	0,53	0,59		0,43	0,39		0.16				

(a) ANOVA; (b) Paired Student’s t test comparing pre of the uncomplicated group with pre of the complicated group; (c) Paired Student’s t test comparing discharge of the uncomplicated group with discharge of the complicated group; (d) Paired Student’s t test comparing six months in the uncomplicated group with six months in the complicated group; (e) Paired Student’s t test comparing discharge from ICU of the uncomplicated group with discharge of the complicated group 6MWT - Six Minute Walk Test; WS - Walking Speed; SST - Sit and Stand Test; TUG - Timed Up and Go; MRC - Medical Research Council; FIM - Functional Independence Measure; MIP - Maximum Inspiratory Pressure; MEP - Maximum Expiratory Pressure; VC - Vital Capacity; PEF - Peak expiratory flow

## Discussion

Based on the observed data from the results, we found that patients with postoperative CABG complications may have reduced functional performance, muscle strength, and pulmonary function at hospital discharge and after six months. Another significant finding was the increased length of hospital stay in the group with complications compared to those without complications.

Cristhine et al. [18] concluded that the pulmonary complication presented by the patients is probably multifactorial in origin, including the presence of atelectasis and hydrostatic pulmonary edema. It also mentions that postoperative pulmonary complications can induce longer hospital stays, influencing hospital expenses. In another study by Beccaria et al., it was stated that pulmonary complications are the second most frequent cause of morbidity and mortality in the postoperative period of cardiac surgery [19].

Patients who present postoperative complications tend to remain bedridden for longer. This factor contributes to decreased pulmonary function, muscle strength, and functional performance. We noticed in the present study that the performance in functional tests was lower in the group that presented complications, corroborating the current evidence.

Performance on the six-minute walk test is influenced by cardiorespiratory and metabolic fitness, so patients with systemic muscle weakness tend to perform worse on this test. The patient’s prolonged time in bed directly interferes with his performance on the TUG and SST tests that are influenced by balance [20]. The results showed that functional alteration is present at hospital discharge and after six months, and may consequently impair their quality of life and social participation, although this type of test was not evaluated in our study.

Evidence shows that the surgical approach causes decreased functional performance and that this loss is associated with post-surgical complications [21]. Due to the aforementioned complications, physiotherapeutic care is essential during this process. In the study by Chen et al. in the preoperative evaluation, the patients were equivalent in relation to physical performance and manual pressure strength, but in the reevaluation 5 days after surgery, there was a significant decrease in muscle strength in the group without physiotherapy intervention [22].

In the evaluation of PEF, we found that there was an increase in values between the two groups after hospital discharge. This result may be associated with the coughing stimulus that is performed during the hospital stay. In the other tests, it was found that the values of MIP, MEP and VC at hospital discharge had decreased in relation to the preoperative period, this decline was higher in the group with complications, but there was recovery after six months, but not reaching the initial values [23].

The timing of the return to preoperative values is not yet clear. Evidence comparing the pulmonary function of patients who remained active after hospital discharge is lacking. Among the groups presented, a higher incidence of complications such as atelectasis, pneumonia, and pneumothorax can be observed, being highlighted as the main postoperative pulmonary complications. In a study by Silva et al. it is stated that CABG can cause complications in the postoperative period in general, but the respiratory system is more affected, and thus lung function will reduce [19].

In our study, we also observed a considerable percentage of arrhythmias and coagulation disorders, being complications that can lead the patient to death. Arrhythmias have proven to be a common complication after cardiothoracic surgery; in a general context, we have an estimate of 11–40% of patients who develop arrhythmias after CABG [22]. Every CABG surgery will have a risk of bleeding due to the cardiopulmonary bypass that causes altered platelet function and intrinsic coagulation systems [23].

Patients who developed postoperative complications had to spend more time in the hospital, thus generating higher hospital costs for requiring specific treatment, besides being exposed to the risk of infections and decreased quality of life.

Our study has some limitations such as the lack of a sample size calculation, no assessment of pain level, and no follow-up of the patient for a longer period.

## Conclusion

Patients with postoperative CABG complications may have reduced functional performance, muscle strength, and lung function at hospital discharge, continuing after six months.

## Data Availability

All data and materials are available for consultation.
